# Minimal *In Vivo* Efficacy of Iminosugars in a Lethal Ebola Virus Guinea Pig Model

**DOI:** 10.1371/journal.pone.0167018

**Published:** 2016-11-23

**Authors:** Joanna L. Miller, Simon G. Spiro, Stuart D. Dowall, Irene Taylor, Antony Rule, Dominic S. Alonzi, Andrew C. Sayce, Edward Wright, Emma M. Bentley, Ruth Thom, Graham Hall, Raymond A. Dwek, Roger Hewson, Nicole Zitzmann

**Affiliations:** 1 Antiviral Research Unit, Oxford Glycobiology Institute, Department of Biochemistry, University of Oxford, South Parks Road, Oxford, OX1 3QU, United Kingdom; 2 Public Health England, Porton Down, Salisbury, United Kingdom; 3 The Royal Veterinary College, London, United Kingdom; 4 Viral Pseudotype Unit, Faculty of Science and Technology, University of Westminster, London, United Kingdom; Division of Clinical Research, UNITED STATES

## Abstract

The antiviral properties of iminosugars have been reported previously *in vitro* and in small animal models against Ebola virus (EBOV); however, their effects have not been tested in larger animal models such as guinea pigs. We tested the iminosugars *N*-butyl-deoxynojirimycin (*N*B-DNJ) and *N*-(9-methoxynonyl)-1deoxynojirimycin (M*O*N-DNJ) for safety in uninfected animals, and for antiviral efficacy in animals infected with a lethal dose of guinea pig adapted EBOV. 1850 mg/kg/day *N*B-DNJ and 120 mg/kg/day M*O*N-DNJ administered intravenously, three times daily, caused no adverse effects and were well tolerated. A pilot study treating infected animals three times within an 8 hour period was promising with 1 of 4 infected *N*B-DNJ treated animals surviving and the remaining three showing improved clinical signs. M*O*N-DNJ showed no protective effects when EBOV-infected guinea pigs were treated. On histopathological examination, animals treated with *N*B-DNJ had reduced lesion severity in liver and spleen. However, a second study, in which *N*B-DNJ was administered at equally-spaced 8 hour intervals, could not confirm drug-associated benefits. Neither was any antiviral effect of iminosugars detected in an EBOV glycoprotein pseudotyped virus assay. Overall, this study provides evidence that *N*B-DNJ and M*O*N-DNJ do not protect guinea pigs from a lethal EBOV-infection at the dose levels and regimens tested. However, the one surviving animal and signs of improvements in three animals of the *N*B-DNJ treated cohort could indicate that *N*B-DNJ at these levels may have a marginal beneficial effect. Future work could be focused on the development of more potent iminosugars.

## Introduction

The 2013–2016 epidemic of Ebola virus disease (EVD) in West Africa has highlighted the paucity of licensed, direct acting antivirals that can be quickly mobilised for use in outbreak situations [1]. With this in mind, we sought to identify whether a licensed drug, Miglustat (*N*-butyl deoxynojirimycin, *N*B-DNJ), could be of use in the treatment of EVD. *N*B-DNJ is an iminosugar, a class of sugar mimics in which a nitrogen atom is substituted for the endocyclic oxygen [2, 3]. As a result, many iminosugars are inhibitors of sugar utilising enzymes, such as glycosidases and glycosyltransferases [4, 5]. Many viral glycoproteins require processing by host α-glucosidases for interaction with the calnexin/calreticultin pathway to allow correct protein folding. As such, iminosugars that have activity against α-glucosidases have repeatedly been shown to have antiviral activity against a wide range of enveloped viruses *in vitro*, including HIV [6–9], hepatitis B virus [10], dengue virus [11], influenza virus [12] and others [13–16]. Importantly for this work, a number of related iminosugars have been shown to be antiviral against Ebola virus (EBOV) *in vitro* and in mouse models [17, 18].

The antiviral effect of iminosugars has been linked to their inhibition of the endoplasmic reticulum (ER) α-glucosidases I & II. These enzymes remove the first two glucose residues from the Glc_3_Man_9_GlcNAc_2_ (Glc, glucose; Man, mannose; GlcNAc, N-acetylglucosamine) glycan which is the prototype glycan added to all suitable *N*-linked glycosylation sites during translocation of the nascent peptide into the ER [19]. The enzymatic activity of the α-glucosidases creates the Glc_1_Man_9_GlcNAc_2_ glycan, which is the substrate for the ER chaperones calnexin and calreticulin [20]. These chaperones retain glycoproteins in the ER and recruit the protein disulphide isomerase ERp57 to facilitate the correct folding of the glycoprotein [21, 22]. The glycoprotein dissociates from calnexin/calreticulin by the time-dependent removal of the final glucose by α-glucosidase II. Subsequently, if the folding sensor UDP-glucose glycosyltransferase 1 (UGGT1) detects that the protein is not in the correct conformation it can replace this glucose allowing reassociation with the chaperones, thus creating a quality control cycle, the calnexin/calreticulin cycle [20, 23]. When cells are treated with α-glucosidase inhibitors, the glycoproteins remain in the Glc_1-3_Man_9_GlcNAc_2_ form, thus preventing quality control by calnexin/calreticulin cycle [20]. This can result in the aberrant folding of certain glycoproteins, robbing them of their function and resulting in the production of non-infectious virions. This has been demonstrated for at least two viral envelope glycoproteins, HIV gp120 [24–26] and bovine viral diarrhoea virus E1/E2 [27]. Despite being a host process, α-glucosidase inhibition is antiviral at much lower concentrations than it is cytotoxic.

There are several reasons to believe that *N*B-DNJ might have efficacy against EBOV. Firstly, EBOV entry is mediated by a trimer formed of the heavily glycosylated, disulphide-linked GP1 and GP2 glycoproteins [28, 29], thus making calnexin/calreticulin interaction highly likely, though not necessarily vital. Although a role for these chaperones in the folding of GP (the GP1/2 polyprotein) has not been directly demonstrated, it has been shown that GP is not incorporated into vesicular stomatitis Indiana virus virions pseudotyped with EBOV GP if both of the *N*-linked glycan sites in the GP2 portion are deleted [30, 31]. As deletion of each glycan site is not individually deleterious, this suggests that GP requires at least one glycan to complete glycoprotein export, and it is possible that this is because it needs at least one glycan to interact with calnexin/calreticulin. Secondly, three deoxynojirimycin (DNJ, the parent molecule of *N*B-DNJ) derivatives, also α-glucosidase inhibitors, have been shown to weakly inhibit the entry of lentiviruses pseudotyped with Ebola glycoproteins [18]. These agents were also able to protect mice challenged with an adapted EBOV strain; however, they are not licensed for human use. Finally, *N*B-DNJ was effective *in vitro* at reducing EBOV replication and another DNJ derivative, M*O*N-DNJ (*N*-(9-methoxynonyl)-1-deoxynojirimycin, UV-4) was able to protect mice from challenge with the virus (US patent application no. 2011/0065754).

*N*B-DNJ was originally developed as a potential antiviral for HIV and, while promising *in vitro*, it showed little activity in phase I and II trials, apparently due to the insufficient plasma concentrations achieved with oral dosing [32, 33]. Oral doses could not be increased because higher doses led to unpleasant gastrointestinal side effects (diarrhoea and flatulence) due to the drug’s off-target inhibition of intestinal disaccharidases. *N*B-DNJ also inhibits ceramide-dependent glucosyltransferase, an activity exploited in its use in substrate reduction therapy for the lysosomal storage disorder, Gaucher’s Disease. *N*B-DNJ is licensed as a Gaucher drug, where it is administered at sub-antiviral concentrations. *N*B-DNJ has been in the clinic for 14 years with no serious adverse event reports beyond those on the original label, lending hope that this class of compounds can be used as antivirals either by finding ways to give higher doses or finding more efficacious iminosugars.

Despite the unsuccessful first HIV trial, we believe that the reasons for this failure are surmountable, and that EVD may be a more suitable candidate for the clinical use of iminosugars than HIV/AIDS. Iminosugars could be administered parenterally to potentially reduce the gastrointestinal side effects of oral dosing as a bolus; this is inappropriate for chronic outpatient conditions like HIV but is much more workable for severe diseases like EVD where patients are likely to be hospitalised for the course of the disease. As EVD is an acute disease, to which acquired immunity eventually develops, extended courses of treatment may not be necessary, making side effects easier to tolerate and manage. In addition, new iminosugars have been developed over the last two decades to provide greater potency against the target enzymes with fewer off-target effects [34–36].

We tested *N*B-DNJ and M*O*N-DNJ for safety in uninfected guinea pigs and for efficacy in the well-established model of guinea pigs infected with an adapted strain of EBOV. We chose *N*B-DNJ as it is already in clinical use in humans, albeit at lower concentrations than required for antiviral effect, and M*O*N-DNJ as it is currently in phase I clinical trials for safety in humans at doses sufficient to potentially achieve antiviral efficacy (NCT02061358 and NCT02696291).

## Methods

### Iminosugars

*N*-butyl-deoxynojirimycin (*N*B-DNJ) and *N*-nonyl-deoxynojirimycin (*N*N-DNJ) were gifts from Oxford Glycosciences, *N*-(9-methoxynonyl) DNJ (M*O*N-DNJ) was a gift from Unither Virology LLC. The deoxygalactonojirimycin (DGJ) mimics of *N*B-DNJ and *N*N-DNJ, *N*B-DGJ and *N*N-DGJ, were from Toronto Research Chemicals. Iminosugars were all endotoxin free and >95% pure as determined by nuclear magnetic resonance or HPLC. *N*B-DNJ was reconstituted in sterile water for all *in vivo* studies. M*O*N-DNJ was formulated for the efficacy studies in acidified water in the form of the hydrochloride salt (*N*-9-methoxynonyl-deoxynojirimycin-HCl), which has a molecular weight approximately 11.4% larger than M*O*N-DNJ base (e.g.120 mg/kg/day of M*O*N-DNJ base formulated as 133.7 mg/kg/day of M*O*N-DNJ salt): all concentrations refer to the M*O*N-DNJ base.

### Virus

EBOV strain 1976/Yambuku-Ecran (previously named ME718) [37] was previously adapted by serial passage in guinea pigs to cause lethality [38]. Virus titres were measured by TCID_50_ assay on VeroE6 cells (European Collection of Cell Cultures, UK) to confirm infectious doses used during *in vivo* studies.

### Guinea pigs

The use of an intravenously cannulated guinea pig model allowed safer sequential treatment in maximum containment level facilities and has been previously described for EBOV-infected animals [39]. Outbred adult female Dunkin-Hartley guinea pigs weighing 250–350 g with cannulas surgically implanted into the right jugular veins were obtained from Charles River Laboratories, France (safety and first efficacy studies) and Harlan Laboratories, UK (second efficacy study). Animals were allowed to recuperate from the stress of transportation for 24 hours before the study began. All procedures were undertaken according to the United Kingdom Animals (Scientific Procedures) Act 1986. Food and sterile water were available *ad libitum*. For animal procedures, guinea pigs were anaesthetised with 1.5% to 2% isofluorane in oxygen in an induction chamber until full sedation was achieved. Animals infected with EBOV were housed within flexible film isolators under climate-control conditions in an animal containment level 4 room. Guinea pigs were treated with intravenous (IV) *N*B-DNJ, M*O*N-DNJ or water three times daily (TID). Individual temperatures (via indwelling temperature chip) weights and were monitored and recorded daily for the duration of each study. Clinical signs were monitored at least twice daily and a numerical score assigned for analysis: 0, healthy; 2, ruffled fur; 3 lethargy, pinched or hunched; 5, rapid breathing; 10 immobile. Guinea pigs were euthanised to prevent unnecessary suffering when a weight loss of 20% of their original weight was recorded or 10% weight loss with another clinical sign. In the safety study animals were humanely euthanized with a rising concentration of carbon dioxide as per schedule 1 of the Welfare of Animals (Scientific Procedures) Act (1986); pentobarbital sodium was avoided to prevent splenic engorgement masking a possible decrease in spleen size caused by the drugs. In subsequent studies, animals were humanely euthanised by intravenous injection of 200 mg/ml pentobarbital sodium due to spleen measurements not being required. All experimental procedures and studies were preapproved and performed according to guidelines set by the Home Office, UK via project licence number PPL 30/3247. Due to the technical constraints of experiments involving live animals infected with EBOV with TID dosing, pre-test power calculations were undertaken to determine treatment group sizes in the second efficacy study. At alpha = 0.05 and power of 0.8, a 2:1:1 ratio of drug:placebo:untreated was calculated to give the greatest chance of statistically significant results with the limitation of a maximum experiment size of 12 animals. Survival data were analysed in GraphPad Prism 6 using log-rank analysis.

### Dose escalation safety study

Treatments were administered in 0.5 ml volumes (single bolus) to the animals via their IV cannulas TID equally spread over an 8 hour period (~9:00, 13:00 and 17:00). *N*B-DNJ was administered at 433 mg/kg/dose for the first 2 days thereafter at 617 mg/kg/dose (1850 mg/kg/day) until day 15. M*O*N-DNJ was administered at sequential doses of 5, 10 and 20 mg/kg/dose on the first day of treatment. On the second day animals received three doses of 30 mg/kg and then from day three they received 40 mg/kg/dose (120 mg/kg/day) for the remaining 14 days. Compounds were prepared daily, based on weights from the previous day, from stocks of 460 mg/ml *N*B-DNJ (2.1 M), pH 6.8, 30 mg/ml M*O*N-DNJ (94 mM), pH 3.0 or water, pH 3.0 (placebo). After 14 days of treatment animals were euthanised and examined *post mortem* for pathology. The spleen and liver were removed from each animal and weighed. Samples were also collected in 10% formalin for oligosaccharide analysis.

### Efficacy studies

Two efficacy studies used different timings of the TID dosing regimen. In the first efficacy study, animals were challenged by sub-cutaneous injection of 10^3^ 50% tissue culture infectious doses (TCID_50_) EBOV (previously shown to cause lethal disease [40]) on day 0, then immediately treated IV TID equally spread over an 8 hour period (~9:00, 13:00 and 17:00) with ~0.5 mL water (pH 6.6), 1850 mg/kg/day *N*B-DNJ (pH 7.0) or 120 mg/kg/day M*O*N-DNJ (pH 6.3) until day 14. In the second efficacy study, animals were challenged with 10^3^ TCID_50_ EBOV on day 0, then immediately treated IV TID (at evenly spaced 8 hour intervals) with ~0.5 mL water (pH 6.1), 1850 mg/kg/day *N*B-DNJ (pH 7.1) or untreated until day 14. Weights and temperatures were recorded daily and clinical signs monitored at least twice a day for the duration of the study on individual animals.

### Histopathology

Samples of liver and spleen were taken from each animal at necropsy and fixed in 10% formalin, processed routinely to paraffin wax, sections cut at 3–5 μm and stained with haematoxylin and eosin (HE). Lesion severity was scored independently by two pathologists.

### Free oligosaccharide analysis

Post mortem liver samples were assayed for the presence of free oligosaccharides (FOS) as previously described by Alonzi et al [41]. Around 30mg (wet weight) of liver was lysed by cycles of freeze-thawing in double-distilled water. Following cell lysis, samples were subjected to mixed-bed ion exchange and then lyophilised. FOS were labelled with 2-aminobenzoic acid (2-AA) and purified using a DPA-6S column (Sigma). Unconjugated 2-AA was removed by phase splitting with ethyl acetate, and samples were lyophilised and resuspended in water then purified using a concanavalin-A column. Glycans were separated by normal phase-high performance liquid chromatography (NP-HPLC) and peak area was used to assess molar quantity in comparison to standards of known identity and quantity. FOS generation was normalised to wet weight.

### qRT-PCR

Blood from Ebola-infected guinea pigs was harvested into RNAprotect tubes and stored at -80°C. RNA was isolated using RNeasy extraction kits (Qiagen). The experimental RNA samples were treated using Turbo DNase kit (Ambion, Life Technologies) and reverse transcribed using High Capacity RNA-to-cDNA kit, as per manufacturer’s guidelines (Applied Biosystems). Real-time PCR was performed to measure gene expression of guinea pig cytokines IFNγ, TNFα, IL-2, IL-12, IL-5, IL-17, TGFβ and endogenous controls β-actin and β-2 microglobulin. The PCR was carried out using primer and probe sequences detailed in S1 Table. A total of 1 ng of RNA from the reverse-transcription reaction was added to Gene Expression PCR mix (Applied Biosystems). A no-template control for each target, reverse transcription negative controls and guinea pig RNA calibrator controls were run alongside the experimental samples. The PCR was performed on an ABI Prism 7900HT Sequence Detection System (Applied Biosystems) as per manufacturer’s guidelines.

### EBOV glycoprotein pseudotyped lentivirus infection and RT activity assays

Ebola virus pseudotypes were produced as previously described [42]. Briefly, 293T/17 cells were transfected with pCMV-Δ8.91, pCSFLW and pCAGGS-EBOV, the latter containing the GP from the Mayinga isolate of Ebola virus (EU224440), in the presence or absence of compound (100 μM). The media was changed 16 hours post-transfection and supernatant harvested 48 hours later. Infection of fresh 293T/17 cells, in the presence or absence of compound (100 μM), enabled viral titres to be determined as measured by the level of reporter gene activity. DMSO and media alone controls were used to establish background levels of infection.

### Statistical analysis

To compare weight differences between groups in the efficacy experiment, the non-parametric Mann-Whitney statistical test was used due to the limited group sizes and data not being normally-distributed. Data were analysed in Minitab version 16.2.2 and were considered statistically significant if p < 0.05.

## Results

### *N*B-DNJ and M*O*N-DNJ were well tolerated in guinea pigs

A toxicity study was conducted in naïve guinea pigs to ensure that they could tolerate delivery of the iminosugar without any adverse effects. Animals were treated with *N*B-DNJ (n = 6), M*O*N-DNJ (n = 4) or water placebo (n = 4) TID. Maximum safely tolerated doses were determined based on previously published data where available. *N*B-DNJ is safe up to 2400 mg/kg/day PO in rodents [43] and within the limitations of allowable injection volume, dosing regimen and solution viscosity we could administer a maximum dose of 1850 mg/kg/day IV. As such, guinea pigs were dosed with just one intermediate concentration, 433 mg/kg TID, for two days after which doses were maintained at 617 mg/kg (the maximum concentration soluble in water) TID for a further 14 days. Due to a lack of published studies specifically looking at the tolerability of M*O*N-DNJ in rodents at the time of the experiment, and informed by *in vitro* observations that M*O*N-DNJ is more toxic than *N*B-DNJ in previously published comparisons [44, 45], a smaller increment dose escalation was performed with this drug. Guinea pigs received sequential doses of 5, 10 and 20 mg/kg M*O*N-DNJ. On the second day they received three doses of 30 mg/kg and then from day three they received 40 mg/kg for the remaining 14 days. Animals were monitored for weight, temperature and clinical health. After 16 days of treatment, animals were euthanised and then necropsied. One animal in the *N*B-DNJ-treatment group seemed to suffer heart failure (cause undetermined) during dosing on day 11 and was humanely culled. Both liver and spleen appeared normal on necropsy and as death was unrelated to compound this animal was not included in the analysis.

**Fig 1:**
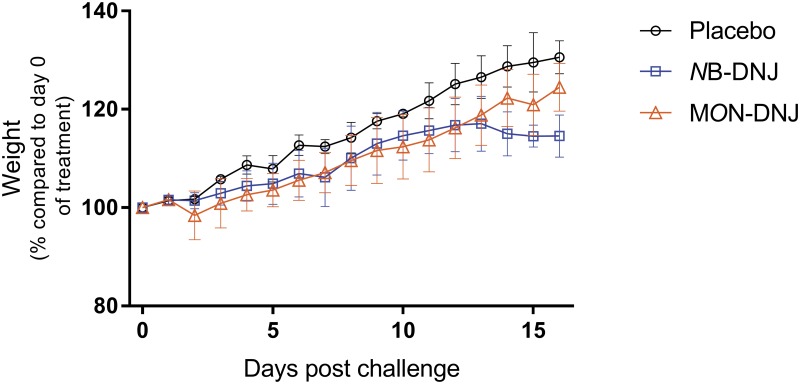
Effect of iminosugar treatment on guinea pig weight. Female guinea pigs were treated via IV cannula TID with 1850 mg/kg/day *N*B-DNJ (n = 6), 120 mg/kg/day M*O*N-DNJ (n = 4) or placebo (n = 4) for 16 days. Weight change as a percentage of body weight on day 0 (baseline) is shown for placebo (black circle), *N*B-DNJ (blue square, including euthanised animal) and M*O*N-DNJ (orange triangle) treatment groups. Means for each group +/- standard deviation are plotted.

*N*B-DNJ treatment resulted in decreased weight gain (Fig 1), as has been seen previously in mice given high doses of *N*B-DNJ [46]. The *N*B-DNJ-treatment group, but not the M*O*N-DNJ-treatment group, had statistically significantly reduced mean weight compared with the placebo-treatment group (p < 0.05, one way ANOVA) on days 14–16. No abnormalities attributable to the treatments were observed in temperature (S1 Fig), clinically (data not shown), histologically (data not shown), in liver and spleen organ mass (S1 Fig) or on necropsy (data not shown). To assess whether the drugs were reaching effective concentrations, post-mortem liver samples were assayed for the presence of free oligosaccharides (FOS), a correlate of ER α-glucosidase inhibition [41]. *N*B-DNJ and M*O*N-DNJ reached sufficient concentrations in the tissues to inhibit ER α-glucosidases as determined by elevated FOS (S2 Fig).

### Equivocal efficacy of *N*B-DNJ in EBOV-infected guinea pigs

To test efficacy of *N*B-DNJ and M*O*N-DNJ against EBOV, guinea pigs were treated IV with TID dosing beginning immediately after EBOV challenge. Untreated animals all met humane clinical endpoints, confirming a lethal dose of EBOV.

**Fig 2:**
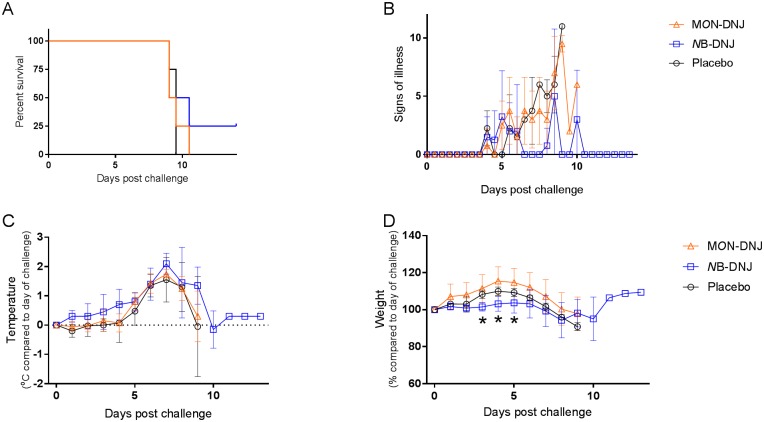
Efficacy of *N*B-DNJ and M*O*N-DNJ in guinea pigs challenged with EBOV. Female guinea pigs were infected with 10^3^ pfu of EBOV (Zaire strain) via IV cannula then treated by the same route TID (between 9am and 5pm) with 1850 mg/kg/day *N*B-DNJ (n = 4), 120 mg/kg/day M*O*N-DNJ (n = 4) or placebo (n = 4) for 14 days. (A) Percentage of surviving animals. (B) Signs of illness. (C) Body temperature, compared to their temperature on day 0 (baseline) and (D) weight change as a percentage of body weight on day 0 (baseline). Mean for remaining animals in each group plotted for B, C and D. Asterisks indicate significantly different weights (p < 0.05; Mann-Whitney test) between animals in placebo and *N*B-DNJ-treated groups at day 3 (p = 0.0304) and in *N*B-DNJ- and M*O*N-DNJ-treated groups at day 4 (p = 0.0304) and day 5 (p = 0.0304).

The first efficacy study dosed animals on day 0 immediately following EBOV challenge, TID with doses equally spread within an ~8 hour period (~ 9:00, 13:00 and 17:00) with 1850 mg/kg/day *N*B-DNJ (n = 4), 120 mg/kg/day M*O*N-DNJ (n = 4) or water placebo (n = 4). Survival analysis showed all animals meeting humane endpoints at days 9–11 post-challenge except for an animal in the *N*B-DNJ treated group which survived until the end of the study (Fig 2A). Animals from all groups began to show clinical signs from day 4 post challenge with those in the placebo and M*O*N-DNJ groups increasing in severity and all animals in these groups were euthanised on day 9–10 post challenge (Fig 2B). Animals treated with *N*B-DNJ showed reduced signs of illness on days 7–8, compared with placebo and M*O*N-DNJ treated animals, even though all animals in the study had elevated temperatures (Fig 2C) indicative of EBOV infection. The reduced weight gain observed in *N*B-DNJ-treated animals in the toxicity study (Fig 1), was also observed in this challenge study (Fig 2D). The weight loss of 1 animal in the *N*B-DNJ group plateaued on days 8–10, then reversed (Fig 2D); this animal had no fever or clinical signs left on day 14 and survived the critical phase of disease (Fig 2A).

### Reduced severity of histopathological lesions in *N*B-DNJ-treated, EBOV challenged guinea pigs

**Fig 3:**
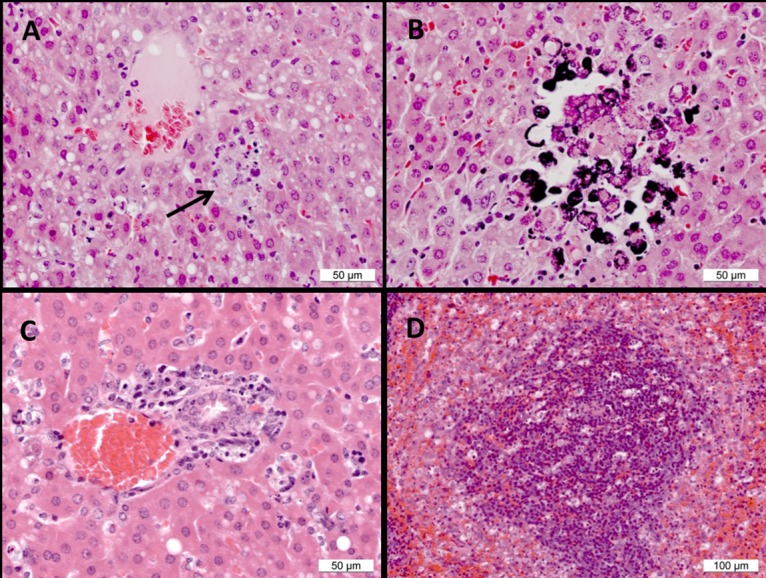
Histopathological observations in Ebola virus-challenge guinea pigs. (A) Liver, placebo animal 1. Hepatocyte vacuolation and a focus of necrosis (arrow). (B) Liver, placebo animal 1. Hepatocyte necrosis and mineralisation of necrotic cells. (C) Liver, M*O*N-DNJ-treated animal 11. Necrosis and depletion of hepatocytes from around a portal triad. (D) Spleen, M*O*N-DNJ-treated animal 10. Scattered necrotic/apoptotic cells and macrophage-like cells in the red pulp at the interface with the marginal zone.

Histopathology showed lesions in the liver consisting of abnormal vacuolation of hepatocytes (Fig 3A), foci of necrosis with mild neutrophilic and/or lymphocytic infiltration (Fig 3A), mineralisation (Fig 3B), necrosis and depletion of hepatocytes from around portal triads and increased numbers of mononuclear cells around portal triads (Fig 3C). In the spleen, abnormalities in the red pulp comprised congestion (Fig 3D), a patchy increase in numbers of polymorphs and diffuse infiltration of macrophages. In the white pulp depletion of lymphocytes from peri-arteriolar lymphoid sheaths, scattered single cell necrosis/apoptosis (Fig 3D) and an increase in the numbers of macrophages at the interface with red pulp (Fig 3D). There was a marked absence of lymphoid follicles in the splenic white pulp of all animals.

Lesions attributable to EBOV infection were detected in animals from all treatment groups but comparisons of placebo-treated animals with animals treated with *N*B-DNJ indicate a degree of reduced severity in *N*B-DNJ-treated animals (Table 1). In *N*B-DNJ-treated animals, eight liver lesions scored as ‘within normal limits’ compared with one in placebo-treated animals. Similarly, in the spleen, fourteen lesions in *N*B-DNJ-treated animals were scored as ‘within normal limits’ compared to none in placebo-treated animals. Treatment with M*O*N-DNJ did not reduce the severity of liver or spleen lesions.

**Table 1:** Severity of histological lesions in HE stained tissues from iminosugar treated guinea pigs, challenged with EBOV.

		Placebo				*N*B-DNJ				M*O*N-DNJ			
		1	2	3	4	5	6	7	8	9	10	11	12
	Lesion/Time of death (Day)	9	9	9	9	10		9	9	9	9	9	10
**Liver**	Hepatocyte vacuolation	Mod	Mod	Min	Mod	MKd	WNL	Mild	Mild	Mkd	Mkd	Mod	Mkd
	Focal necrosis	Mod	Min	Mkd	Mod	Mod	WNL	Mild	Mod	Mkd	Mkd	Mkd	Mkd
	Focal mineralisation	Mod	WNL	Mkd	Mod	Mild	WNL	WNL	Min	Mkd	Mkd	Mod	Mkd
	Depletion of hepatocytes from portal triads	Mod	Min	Min	Min	Min	WNL	Min	Mod	Mod	Min	Mod	Mod
	Infiltration of portal areas by macrophages/fibroblasts	Mod	Min	Min	Min	Min	WNL	WNL	WNL	Mild	Min	Mod	Mild
**Splenic red pulp**	Congestion	Mod	Mod	Mkd	Mkd	Mod	WNL	Mild	Mild	Mod	Mkd	Mod	Mild
	Scattered single cell necrosis	Mod	Mkd	Mkd	Min	Mod	WNL	WNL	WNL	Mod	Mod	Min	Min
	Patchy polymorph infiltration	Mod	Mkd	Mkd	Mkd	Mod	WNL	WNL	WNL	Mod	Mild	WNL	WNL
	Diffuse infiltration of macrophages	Mod	Mild	Mod	Mod	Mod	WNL	Min	Mild	Mod	Mod	Mod	WNL
**Splenic white pulp**	Scattered single cell necrosis	Mod	Mkd	Mkd	Mild	Mild	WNL	Mild	WNL	Mod	Mod	Min	Min
	Lymphocyte depletion	Mod	Mod	Mod	Mod	Mod	WNL	WNL	Mild	Mod	Mkd	Mod	Min
	Increased numbers of macrophages at interface with red pulp	Mild	Mild	Mild	Mild	Min	WNL	WNL	Mod	Mild	Mod	Min	WNL

WNL (within normal limits, dark blue) and Min (minimal, light blue), Mild (white), Mod (moderate, pink) and Mkd (marked, red). Guinea pig 6 survived until the end of the experiment, day 14.

### Iminosugar treatment had no significant effect on blood cytokine levels in EBOV-infected guinea pigs

**Fig 4:**
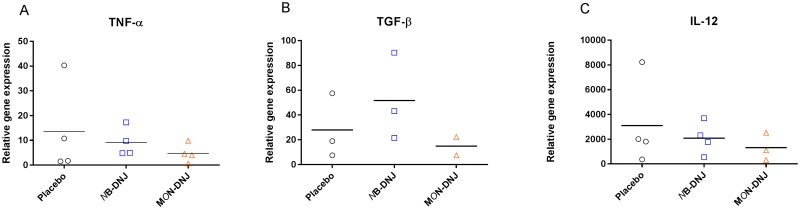
Effect of iminosugars on blood cytokine levels at necropsy. Following infection with EBOV and treatment IV TID with 1850 mg/kg/day *N*B-DNJ, 120 mg/kg/day M*O*N-DNJ or water placebo until euthanised (day 9 or 10 for all animals except one in the *N*B-DNJ treatment group which survived until day 14), blood taken at necropsy was analysed for (A) TNFα, (B) TGFβ and (C) IL-12 mRNA levels by qRT-PCR. Specific cytokine mRNA levels were normalised to β2 microglobulin RNA level and plotted as individual animals with a bar for mean relative gene expression within each group (2–4 animals per group). There were no significant differences between the effects of either of the treatments and the placebo (1-way ANOVA, p = 0.05).

Blood taken at necropsy was assayed by qRT-PCR for guinea pig cytokine mRNA levels to determine whether iminosugar treatment resulted in any impact on the immune response to EBOV. Detectable mRNA was only measured for TNFα, TGFβ and IL-12 and no significant differences were observed between iminosugar-treated and untreated groups (Fig 4). No IFNγ, IL-17, IL-2 or IL-5 mRNA was detected in any of the necropsy samples (data not shown).

### Regular *N*B-DNJ treatment had no effect on survival in EBOV-infected guinea pigs

The half-life of *N*B-DNJ in rodents is short (0.68 hr following IV administration in mice, personal communication, P. Laing; 1.34 hr following oral administration in mice [47]) and in the first efficacy study the plasma concentrations would have dropped to their lowest levels in the guinea pigs overnight, in the 16 hr period between doses. A temporally even dosing regimen or administration by infusion would be expected to result in a more consistent drug plasma concentration which may bring *N*B-DNJ concentrations within the therapeutic window. To investigate whether consistently spaced drug administration could improve survival outcomes a second efficacy study tested the same dose of *N*B-DNJ (n = 6) with uniformly spaced 8 hour dosing intervals. The increased time between doses during the day likely benefited the animals allowing them more time to recuperate between each dose, decreasing stress and thus diminishing the negative effects of cortisol on immune function. Since 120 mg/kg/day M*O*N-DNJ did not confer enhanced survival or decreased clinical signs in the first study, it was not included in the second efficacy study.

In the second efficacy study, guinea pigs were treated IV with TID dosing beginning immediately after EBOV challenge. Animals were dosed TID (every 8 hours), with 1850 mg/kg/day *N*B-DNJ (n = 6) or water placebo (n = 3), following EBOV challenge. Three untreated, EBOV-infected, animals were included as controls for comparison, to examine whether there was any benefit attributable to the osmotic resuscitation by fluids given as placebo treatment. Animals were observed for up to 14 days for morbidity and mortality, when all remaining animals were killed humanely and liver and spleen samples collected for histology.

**Fig 5:**
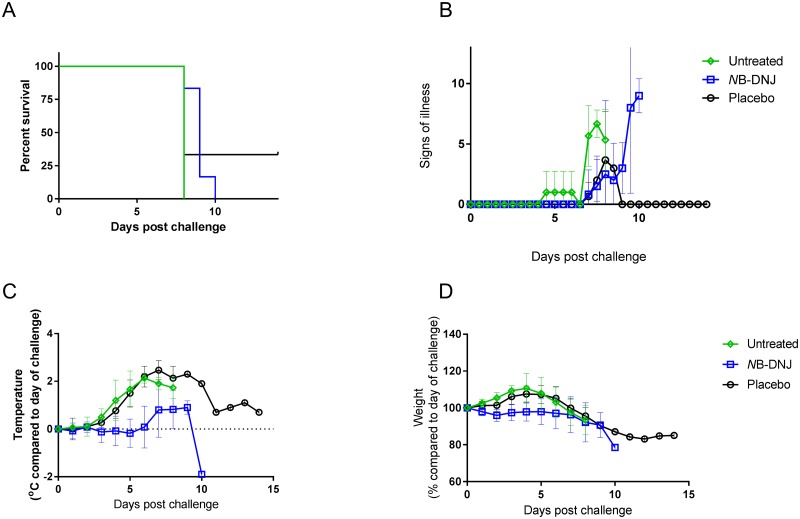
Efficacy of *N*B-DNJ in guinea pigs challenged with EBOV. Female guinea pigs were infected with 10^3^ pfu of EBOV (Zaire strain) via IV cannula. Animals were untreated (n = 3), treated IV TID (8 hourly) with 1850 mg/kg/day *N*B-DNJ (n = 6) or placebo (n = 3) for 14 days. (A) Percentage of surviving animals. (B) Signs of illness. (C) Body temperature, compared to their temperature on day 0 (baseline) and (D) weight change as a percentage of body weight on day 0 (baseline). Mean for each group +/- standard deviation is plotted. Note: Temperatures in panel C show means of only 5 out of the 6 animals treated with *N*B-DNJ due to the temperature chip failing in one of the animals in this group.

One animal survived until day 14, in the placebo treatment group (Fig 5A), despite complete lethality of placebo or untreated animals in previous studies. This animal had lost 17% of its starting body weight by day 12 and had an elevated temperature consistent with EBOV infection, which was confirmed by qRT-PCR at necropsy (data not shown). While all 6 animals treated with *N*B-DNJ reached humane end points and were euthanized, this group demonstrated reduced clinical signs in comparison with untreated animals (Fig 5B) (though no difference to animals treated with placebo), delayed onset of fever (Fig 5C) and, as seen in the initial safety study, reduced weight gain (Fig 5D).

### Similar histopathological changes were present in regularly *N*B-DNJ-treated guinea pigs compared to controls

While significant changes were detected in the liver and spleen of animals from all groups (Table 2), the overall severity of lesions in the second EBOV challenge study was reduced in comparison with those in the previous study. There was a marked absence of lymphoid follicles in the splenic white pulp of all animals; however, other changes in the spleen were at most moderate in all treatment groups. Lesions associated with EBOV were detected in all three groups with no substantial difference in lesion severity in liver and spleen between the three treatments.

**Table 2:** Severity of histological lesions in samples of EBOV challenged animals after treatment (second efficacy study).

	Lesion	Placebo			*N*B-DNJ						Untreated		
		1	2	3	4	5	6	7	8	9	10	11	12
**Liver**	Hepatocyte vacuolation	Mod	WNL	Min	Mkd	Mkd	WNL	Min	WNL	Mkd	Mild	Mkd	Mild
	Focal necrosis	Mild	Min	Mild	WNL	Min	Min	Min	Mod	Mod	Mod	Mod	Mkd
	Focal mineralisation	Min	Mod	WNL	WNL	WNL	WNL	WNL	WNL	WNL	WNL	Mod	WNL
**Splenic red pulp**	Congestion	Mod	Min	Mod	Mod	Mod	Mild	Mod	Mild	Mild	Mod	Min	Mild
	Scattered single cell necrosis	Mod	WNL	Mod	Mod	Mild	Mod	Mild	Mod	Min	WNL	Min	Min
	Patchy polymorph infiltration	Min	Mod	Min	Mild	Min	Mod	Mod	Min	Min	WNL	Min	Min
**Splenic white pulp**	Scattered single cell necrosis	Min	WNL	Mod	Mild	Mild	Mild	Min	Min	WNL	Min	Min	Min
	Lymphocyte depletion	Mild	Mild	Mild	Mild	Mild	Mild	Min	WNL	Mod	WNL	Min	WNL

WNL (within normal limits, dark blue) and Min (minimal, light blue), Mild (white), Mod (moderate, pink) and Mkd (marked, red). Guinea pig 2 survived until day 14.

### Iminosugars did not affect infectivity of EBOV glycoprotein pseudotyped lentivirus

**Fig 6:**
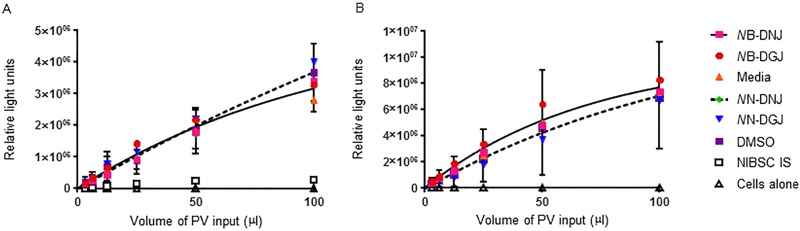
Production of EBOV glycoprotein pseudotyped virus in the presence or absence of iminosugars does not alter infectivity. EBOV glycoprotein pseudotyped lentivirus was produced in the presence of 100μM of *N*B-DNJ, *N*N-DNJ, *N*B-DGJ or *N*N-DGJ, equivalent DMSO or media alone. The infectivity of these viruses was then assayed in HEK 293T cells in the (A) presence or (B) absence of homologous drug and recorded as luciferase reporter gene expression. The NIBSC international serological standard was included as a positive control for neutralisation. Pseudotyped virus production was undertaken twice, each time infections were performed in duplicate, and data analysed together. Average RLU are shown. For clarity the SD error is shown for only media and DMSO data but these are representative of all data sets. Single one phase exponential curves were fitted to the *N*B-DNJ, *N*B-DGJ and media data (solid line) and to *N*N-DNJ, *N*N-DGJ and DMSO data (dashed line).

We examined whether iminosugars could affect infectivity of a lentivirus pseudotyped with EBOV glycoprotein, by producing Ebola pseudotyped virus in the presence of either 100 μM *N*B-DNJ or *N*N-DNJ. The levels of infectious virus were then quantified in HEK 293T cells, both in the presence and absence of 100 μM iminosugar. A single one phase exponential curve could be used to describe the treatments in the presence or absence of iminosugars, excluding the negative control of cells alone and positive inhibition control of the NIBSC international serological standard. Iminosugars with an *N*-butyl head group were compared with media alone control as stocks were dissolved in water (pseudotyped virus produced in the presence of iminosugar: F = 2.491; p = 0.0515; pseudotyped virus produced in the absence of iminosugar: F = 0.4449; p = 0.7757); iminosugars with an *N*-nonyl head group were dissolved in DMSO hence were compared with equivalent DMSO control (pseudotyped virus produced in the presence of iminosugar: F = 1.521; p = 0.2061; pseudotyped virus produced in the absence of iminosugar: F = 0.5032; p = 0.7335). Fig 6 shows that neither *N*B-DNJ nor *N*N-DNJ alter the infectivity of Ebola pseudotyped virus in comparison to virus produced in the presence of media or DMSO alone. The control DGJ analogues also tested do not inhibit α-glucosidases and hence, as expected did not have any effect on pseudotyped virus infectivity. The inclusion of iminosugars during the assay for infectivity did not alter the lack of effect on pseudotyped virus infectivity, thus *N*B-DNJ and *N*N-DNJ do not affect pseudotyped virus infectivity.

## Discussion

The objective of this study was to evaluate iminosugars as treatment for EVD in a guinea pig model. This study was able to show that both *N*B-DNJ and M*O*N-DNJ were safe and well tolerated, even at high doses, in guinea pigs. Subsequent to these experiments being performed, M*O*N-DNJ was also shown to be tolerable via oral dosing in mice up to 150 mg/kg TID for 7 days [48], which can be extrapolated with body surface adjustment to be ~56 mg/kg in guinea pigs. Both drugs were able to inhibit ER α-glucosidases *in vivo*, as demonstrated by the detection of FOS in liver samples taken at the end of the experiment. The initial efficacy study was promising, with 1 of 4 infected *N*B-DNJ treated animals surviving and the remaining three showing improved clinical signs and reduced EBOV-associated lesions by histopathology, although time to death was not affected.

While EVD animal models are not uniformally lethal, and hence the survival of a single animal may not necessarily have warranted it, the improvement in some clinical and histology parameters in all *N*B-DNJ-treated animals was cause for further investigation. However, a second study could not confirm drug-associated benefits. The main difference between the two studies was the timing of drug administration and the source of the animals. It is conceivable that the closer drug administration spacing during day time in the first study led to a higher peak drug plasma concentration which, despite the corresponding lower overnight drug levels, may have been sufficient to lead to an overall improved outcome in this study treatment group. If so, this would suggest that a higher peak concentration, rather than more consistent concentration of iminosugars is more beneficial in this model. The survival of one of the placebo treated animals in the second study suggests that this model is not 100% lethal and/or that the fluid volume in which the drug was given was itself mildly therapeutic. However, the placebo treated animals in the second study did not see the same decrease in clinical scores that the *N*B-DNJ treated animals in the first study did, making the fluid replacement explanation unlikely. As guinea pigs are a relatively outbred population (compared to other laboratory rodents) it may be that the animals responded heterogeneously to both the virus and the drug. Although this study was optimised to provide the highest power given technical constraints, survival of 40% of drug treated animals would have been necessary to achieve statistical significance at a 2:1 drug:placebo control in the absence of untreated animals given the limited sample size. As such, lack of statistically detectable effect may fall within the stochastic nature of the treatment effect for *N*B-DNJ.

In understanding how iminosugars mediate their antiviral effect it is important to take into account both inhibition of host ER α-glucosidases and the amplification hypothesis. Both drugs were able to inhibit ER α-glucosidase II *in vivo*, although only *N*B-DNJ inhibited α-glucosidase I. This effect is probably due to the 15x higher dose of *N*B-DNJ that was administered, and given that M*O*N-DNJ is only 3–4 fold more potent than *N*B-DNJ against α-glucosidase I and α-glucosidase II *in vitro* ([36, 47, 49]), this may explain why *N*B-DNJ showed some effect yet M*O*N-DNJ did not. This also could imply that inhibition of ER α-glucosidase I is needed to achieve an antiviral effect against EBOV. It has been hypothesised that a few misfolded envelope glycoproteins may be sufficient to disrupt viral attachment and/or assembly, and in this way, the effect of iminosugars on virus amplified [16]. In the case of HIV envelope, gp120, the level of misfolding necessary for an antiviral effect was quantified showing that a small decrease in correctly folded gp120 was sufficient to reduce infectivity. While each HIV virion may have as few as 15 gp120 trimers [50], EBOV has more than 100 GP spike proteins per virion [51] suggesting that iminosugar-mediated misfolding of viral glycoproteins may be more challenging against EBOV.

A controlled early immune response is required for survival to infection with EBOV [52]. As the first efficacy study suggested some reduction in disease severity we assayed blood by qRT-PCR for mRNA levels of a number of cytokines relevant to EVD, however iminosugars had no significant effects on the cytokines detected. The lack of IFNγ is unexpected based on a number of other lethal models of EBOV infection but could be a result of the samples being taken at day 9 on necropsy at which time the IFNγ response may have subsided, or due to insufficient immune cells in circulation due to sequestration at local sites of disease or EBOV immunoevasion (viral protein (VP24) has been reported to desensitize the expression of IFNγ in host cells [53]). Positive controls were included in the qRT-PCR. The lack of detection of cytokines produced by T cells in this study has also been reported by Wauquier *et al*., where a decrease in these circulating cytokines was reported with a poor outcome following infection with Ebola in human populations [54].

Our findings are in contrast to murine data from Chang *et al*. [18] who assessed the efficacy of three α-glucosidase inhibitors against EBOV. These compounds were able to inhibit the maturation of GP pseudotyped HIV and were able to protect mice from an adapted strain of EBOV. The difference in efficacy of iminosugars in EBOV-infected mouse and guinea pig models may be due to differences between species, EBOV-strains used or the potency of the different iminosugars, and cannot be determined without performing direct comparisons. Discrepancies in efficacy testing chloroquine against EBOV have been reported previously with efficacy observed in mice [55] but not when tested in guinea pigs [56] or hamsters [57]. The different effects on the pseudotyped virus could similarly be explained by use of GP from different EBOV-isolates and it is worth noting that, in Chang *et al*. the Ebola GP pseudotypes show only modest inhibition by iminosugars, showing at most a 1.5 log decrease in titre by one iminosugar and <0.5 log for the others (cf. 2.5 for bovine viral diarrhoea virus, 3 for dengue virus and 3.5 for Rift Valley fever virus) at 100 μM, the highest concentration tested. Further to this, Chang *et al*. use 293T cells whereas this study uses 293T/17 cells, a clone selected for its ability to express proteins in greater quantities. Therefore, as the effect seen by Chang *et al*. is moderate it could be that the cellular systems involved in GP maturation are more easily overcome in standard 293T cells and the weak effect of the compounds can be observed.

Pharmacodynamic differences between mice and guinea pigs, and between different iminosugars, may be the most likely explanation for the variation in the literature and between our own experiments. Additional studies could be performed to determine whether the different routes of compound administration affected efficacy differences between the published mouse [58] and our guinea pig experiments. The major differences between the two efficacy experiments in this study were the changes in the timing of doses and an unavoidable change in the source of animals. As guinea pigs are outbred it may be that the second cohort were of a different genetic background to the first, and this may have resulted in a different response to the virus and/or drug. The changes in dosing are likely to affect the pharmacodynamics of the drug; indeed, it was our intention that, by spacing out the doses more evenly, we would increase the trough drug concentration, resulting in increased survival. However, this change appeared to decrease the efficacy of *N*B-DNJ. One possible explanation for this may be that while the closely spaced dosing in the first study led to lower plasma concentrations overnight, it may have permitted accumulation to higher and potentially efficacious peak levels during the day. However, plasma concentrations were not measured in this study so this remains hypothetical. *N*B-DNJ and M*O*N-DNJ are small molecules and redistribute quickly with a high volume of distribution, much like the sugars they mimic [59, 60]. Thus dosing IV may have led to a more rapid clearance of the drugs than that seen with the intraperitoneal injections used in previous mouse studies, where the abdominal cavity may have acted as a reservoir to provide a slower infusion of drug into the circulation.

Iminosugars may have efficacy against EBOV in certain contexts but the evidence presented in this study did not show substantial benefits *in vivo* nor any effect in a pseudotype virus system and confirms the value of performing studies in a cogent animal model of infection. The most cogent animal model to be predictive in humans may vary depending on the intervention being tested, with rodents and non-human primates demonstrating inconsistent reciprocal protection in some cases [61]. Other DNJ-derivative iminosugars, such as IHVR11029, IHVR17028 and IHVR19029 [58] have previously demonstrated survival benefits in the EBOV mouse model and hence are worth further study. The 2013–2016 epidemic of EVD invigorated research; however, there are still no licenced antiviral products to treat acute or persistent EVD and, in the event of another outbreak, having published results appear in the public domain will be valuable to health-care providers.

## Supporting Information

S1 Fig *N*B-DNJ and M*O*N-DNJ are safe in guinea pigs. Female guinea pigs were treated IV TID with 1850 mg/kg/day *N*B-DNJ (n = 6), 120 mg/kg/day M*O*N-DNJ (n = 4) or placebo (n = 4) for 16 days. (A) Body temperature, compared to their temperature on day 0 (baseline), mean for each group +/- standard deviation. (B) Organ mass of liver and spleen of each animal at post mortem as a percentage of body weight. Groups were analysed using two-way ANOVA at an alpha of 0.05; no significant differences were observed between treatment groups.
(DOCX)

S2 Fig *In vivo* glycan analysis in guinea pigs treated with iminosugars. Liver samples were obtained at day 16 from guinea pigs treated IV TID with 1850 mg/kg/day *N*B-DNJ (n = 2), 120 mg/kg/day M*O*N-DNJ (n = 3) or placebo (n = 3) for 16 days. FOS was analysed for the presence of (A) Glc_1_Man_4_GlcNAc_1_ as representative of α-glucosidase II inhibition and (B) Glc_3_Man_5_GlcNAc_1_ as representative of α-glucosidase I inhibition.
(DOCX)

S1 Table Primer and probe sequences for PCR of guinea pig cytokine mRNA. (DOCX)
