# Supplementary material for: Minimal In Vivo Efficacy of Iminosugars in a Lethal Ebola Virus Guinea Pig Model
Source: PLoS One. 2016 Nov 23;11(11):e0167018. doi: 10.1371/journal.pone.0167018 (PMC5120828; doi:10.1371/journal.pone.0167018)
Supplement: S1 Fig — Female guinea pigs were treated IV TID with 1850 mg/kg/day NB-DNJ (n = 6), 120 mg/kg/day MON-DNJ (n = 4) or placebo (n = 4) for 16 days. (A) Body temperature, compared to their temperature on day 0 (baseline), mean for each group +/- standard deviation. (B) Organ mass of liver and spleen of each animal at post mortem as a percentage of body weight. Groups were analysed using two-way ANOVA at an alpha of 0.05; no significant differences were observed between treatment groups. (DOCX) [file pone.0167018.s001.docx]

**S1 Fig. *N*B-DNJ and M*O*N-DNJ are safe in guinea pigs.** Female guinea pigs were treated IV TID with 1850 mg/kg/day *N*B-DNJ (n=6), 120 mg/kg/day M*O*N-DNJ (n=4) or placebo (n=4) for 16 days. (A) Body temperature, compared to their temperature on day 0 (baseline), mean for each group +/- standard deviation. (B) Organ mass of liver and spleen of each animal at post mortem as a percentage of body weight. Data from the euthanised animal in the *N*B-DNJ-treatment group is included. Groups were analysed using two-way ANOVA at an alpha of 0.05; no significant differences were observed between treatment groups.

**A**

**B**
