# Supplementary material for: Minimal In Vivo Efficacy of Iminosugars in a Lethal Ebola Virus Guinea Pig Model
Source: PLoS One. 2016 Nov 23;11(11):e0167018. doi: 10.1371/journal.pone.0167018 (PMC5120828; doi:10.1371/journal.pone.0167018)

**S2 Fig. *In vivo* glycan analysis in guinea pigs treated with iminosugars.** Liver samples were obtained at day 16 from guinea pigs treated IV TID with 1850 mg/kg/day *N*B-DNJ (n=2), 120 mg/kg/day M*O*N-DNJ (n=3) or placebo (n=3) for 16 days. FOS was analysed for the presence of (A) Glc_1_Man_4_GlcNAc_1_ as representative of α-glucosidase II inhibition and (B) Glc_3_Man_5_GlcNAc_1_ as representative of α-glucosidase I inhibition.


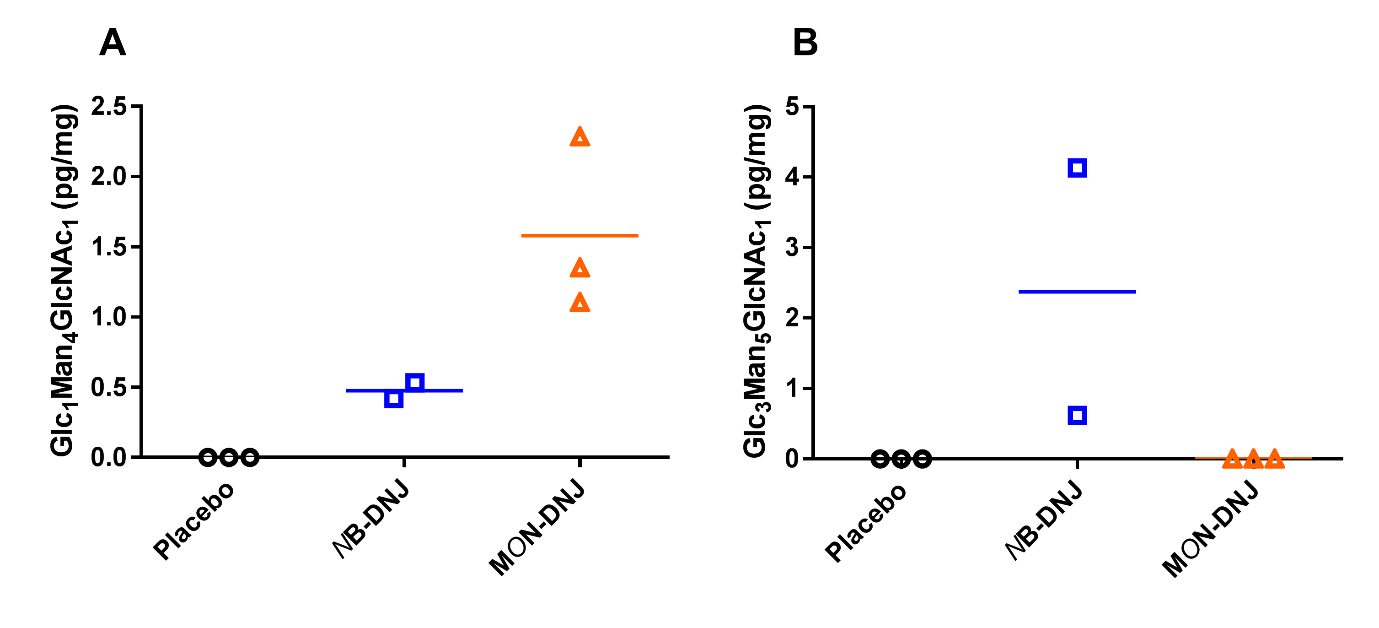

Supplement: S2 Fig — Liver samples were obtained at day 16 from guinea pigs treated IV TID with 1850 mg/kg/day NB-DNJ (n = 2), 120 mg/kg/day MON-DNJ (n = 3) or placebo (n = 3) for 16 days. FOS was analysed for the presence of (A) Glc1Man4GlcNAc1 as representative of α-glucosidase II inhibition and (B) Glc3Man5GlcNAc1 as representative of α-glucosidase I inhibition. (DOCX) [file pone.0167018.s002.docx]
