# Supplementary material for: Minimal In Vivo Efficacy of Iminosugars in a Lethal Ebola Virus Guinea Pig Model
Source: PLoS One. 2016 Nov 23;11(11):e0167018. doi: 10.1371/journal.pone.0167018 (PMC5120828; doi:10.1371/journal.pone.0167018)
Supplement: S1 Table — (DOCX) [file pone.0167018.s003.docx]

**S1 Table: Primer and probe sequences for PCR of guinea pig cytokine mRNA.**

| **Target** | **Accession ID** | **Forward: 5’ → 3’** | **Reverse: 5’ → 3’** | **Probe: 5’ 6-FAM → BHQ1 3’** |
| --- | --- | --- | --- | --- |
| IFNγ | AY151287.1 | ACAAGGTGCAGGCTTTCAAAA | CGGTGACAGGTCATCTATCACTTT | TCAATGACGAGCATGTCCAGCGC |
| TNFα | U77036.1 | CCAGTTCCTTGTGCCACTCA | CCCTAATTCCCTTTCTGAACCA | TCCTTCCGGTTGGGTCCCCTCA |
| IL2 | NM_001172837.1 | CGAGCAGTGCACCTACTTCAAG | GAGTGTCTGTAAATCCCTCAGTAATAGC | CTCCAAAGCAAACACAGGACCGACTGG |
| IL5 | NM_001172970.1 | AGGAGCGACGGAGAGTGAAG | CCGTGTTTATGACAGCAAGAAATT | AGTTCCTGGATTACCTGCA |
| IL12 | AB025724.1 | CAGACCGGAAAAGTGATATCTTAGG | GTATCCTCCAGCATCTTCAAACTCT | TCTGGTAAAACCCTCACCATCCAGGTCA |
| IL17 | NM_001278768.1 | TCCCCATCCAGCAAGAGATC | GGAAAGCGGGCAGTTCTGA | TCCTGCAGAGGGAAC |
| TGFβ | AF191297.1 | GCCAGCCTCGGGACTGT | CCGCTTCACCAGCTCCATAT | ACCTGCAAGACCATC |
| B2M | NM_001172856.1 | TTCAGCAAGGACTGGACTTTCTATC | CTGCAAGAATATTCGTCACTGTCA | CCTGGTGCATGCTGCCTTTACACCC |
| β-actin | AF508792.1 | TTGGCACCCAGCACAATG | CCGCCGATCCACACAGA | ATCATTGCTCCCCCTGAGCGCAA |
